# Murine models of orthopedic infection featuring *Staphylococcus* *aureus* biofilm

**DOI:** 10.5194/jbji-8-81-2023

**Published:** 2023-03-07

**Authors:** Aiken Dao, Alexandra K. O'Donohue, Emily R. Vasiljevski, Justin D. Bobyn, David G. Little, Aaron Schindeler

**Affiliations:** 1 Orthopaedic Research and Biotechnology Unit, the Children's Hospital at Westmead, Westmead, NSW, Australia; 2 The Children's Hospital at Westmead Clinical School, Faculty of Medicine and Health, University of Sydney, Sydney, NSW, Australia; 3 Bioengineering & Molecular Medicine Laboratory, the Westmead Institute for Medical Research, Westmead, NSW, Australia

## Abstract

**Introduction**: Osteomyelitis remains a major clinical challenge. Many published rodent fracture infection models are costly compared with murine models for rapid screening and proof-of-concept studies. We aimed to develop a dependable and cost-effective murine bone infection model that mimics bacterial bone infections associated with biofilm and metal implants. **Methods**: Tibial drilled hole (TDH) and needle insertion surgery (NIS) infection models were compared in C57BL/6 mice (female, N=150 ). Metal pins were inserted selectively into the medullary canal adjacent to the defect sites on the metaphysis. Free *Staphylococcus aureus* (ATCC 12600) or biofilm suspension (ATCC 25923) was locally inoculated. Animals were monitored for physiological or radiographic evidence of infection without prophylactic antibiotics for up to 14 d. At the end point, bone swabs, soft-tissue biopsies, and metal pins were taken for cultures. X-ray and micro-CT scans were performed along with histology analysis. **Results**: TDH and NIS both achieved a 100 % infection rate in tibiae when a metal implant was present with injection of free bacteria. In the absence of an implant, inoculation with a bacterial biofilm still induced a 40 %–50 % infection rate. In contrast, freely suspended bacteria and no implant consistently showed lower or negligible infection rates. Micro-CT analysis confirmed that biofilm infection caused local bone loss even without a metal implant as a nidus. Although a metal surface permissive for biofilm formation is impermeable to create progressive bone infections in animal models, the metal implant can be dismissed if a bacterial biofilm is used. **Conclusion**: These models have a high potential utility for modeling surgery-related osteomyelitis, with NIS being simpler to perform than TDH.

## 1 Introduction

Open fractures, surgical implantation, and prosthetic joint replacements have high associated comorbidity of osteomyelitis (Zimmerli, 2014). While a range of bacterial pathogens can result in bone infection, *Staphylococcus aureus* is the most prevalent cause of osteomyelitis and postsurgical infection (Brinkman et al., 2019). Prophylactic antibiotics are not always effective due to the widespread and increasing incidence of antimicrobial resistance (Urish and Cassat, 2020). Surgical site infection in orthopedic implants has a high disease burden, with high rates of hospitalization and treatment costs (Shah et al., 2017). Clinical trials are the gold standard for assessing the efficacy of interventions to minimize the impact of orthopedic infection. However, such trials can be expensive, difficult to coordinate, and require large patient numbers (Reizner et al., 2014). In contrast, preclinical rodent models represent a valuable first-pass research tool to screen new therapies (Schindeler et al., 2018). While rat models can have more relevant biomechanical outcomes, murine (mouse) models are more economical and accessible. Despite a range of established models for bone healing (Schindeler et al., 2018), there remain no universally accepted preclinical protocols for modeling orthopedic trauma and combined infection. Moreover, models of bacterial inoculation in mice (Windolf et al., 2014, 2013), rats (Mills et al., 2018, 2020), and sheep (Boot et al., 2021) all typically recapitulate implant-associated infection.

We aimed to iteratively develop and test orthopedic infection models in mice with a focus on localized bone defects. This was framed in the context of investigating the role of bacterial biofilm – the layer of microbial cells and extracellular matrix that forms on solid surfaces (Rodríguez-Merchán et al., 2021). Our first research question was the importance of implants and implant surfaces in the establishment of a persistent bone infection. Our second research question was to compare the impact of inoculation with a suspension of free bacteria vs. bacteria grown as part of a biofilm. Ultimately, this work sought to establish reproducible and detailed protocols for implant-containing and implant-free bone infection models in mice.

## 2 Methods

### 2.1 Bacterial and biofilm culture

Two strains of *S. aureus* were sourced from the American Type Culture Collection: ATCC 12600 (used in prior rat infection models) (Mills et al., 2018, 2020) and a high-biofilm-forming strain (ATCC 25923). Bacteria suspensions were prepared by overnight growth of a single colony, with bacterial numbers calculated from spectrophotometry (Cary 300 UV–Vis, Agilent, Las Vegas, NV) with an OD ${}_{600}$ (optical density at 600 nm) of 1 representing $1\times 10^{9}$ colony-forming units (cfu) per milliliter (mL). For inoculation, bacteria were resuspended in injectable saline (0.9 % sodium chloride) and dosed at 10 ${}^{6}$ cfu mL ${}^{-1}$ (in 200 µ L for systemic injection) or $1\times 10^{5}$ cfu (in 5 µ L for local injection). For biofilm preparation, single colonies were picked for culture in tryptic soya broth (TSB) with 20 % glucose in a six-well plate 1 week prior to surgical inoculation. A piece of sterile stainless-steel foil (1 mm ×1 mm) was added to the culture media (2 mL of TSB +20% glucose) to provide a metal surface for biofilm formation, and media was refreshed thrice per week. For inoculation, biofilm was removed from the stainless-steel foil surface with a sterile swab, triturated in saline, and dosed at $1\times 10^{5}$ cfu (in 5 µ L for local injection).

### 2.2 Animal husbandry and ethics

Female C57BL/6 mice (8–12 weeks old) were purchased from Australian BioResources (Moss Vale, NSW, Australia) or the Animal Resources Centre (Canning Vale, WA, Australia), co-housed up to five per cage with access to food and water ad libitum, and allowed to acclimatize for at least 1 week prior to surgery. The study was approved by the local animal ethics committee (K339) and carried out in accordance with the Australian code for the care and use of animals for scientific purposes (2013).

**Table 1:** The study plan of TDH and NIS bone infection models.

Group	Study	Surgery	Site	Injection	N
1	1A	TDH (no pin)	Metaphysis	Sterile saline	5
2	1A	TDH (no pin)	Diaphysis	Sterile saline	5
3	1A	TDH (no pin)	Metaphysis	Local (10 ${}^{5}$ cfu *S. aureus*)	5
4	1A	TDH (no pin)	Diaphysis	Local (10 ${}^{5}$ cfu *S. aureus*)	5
5	1A	TDH (no pin)	Metaphysis	Systemic (10 ${}^{6}$ cfu *S. aureus*)	5
6	1A	TDH (no pin)	Diaphysis	Systemic (10 ${}^{6}$ cfu *S. aureus*)	5
7	1B	TDH-pin	Metaphysis	Sterile saline	5
8	1B	TDH (no pin)	Metaphysis	*S. aureus* (ATCC 12600) (10 ${}^{5}$ cfu)	5
9	1B	TDH-pin	Metaphysis	*S. aureus* (ATCC 12600) (10 ${}^{5}$ cfu)	5
10	1B	NIS-pin	Metaphysis	*S. aureus* (ATCC 12600) (10 ${}^{5}$ cfu)	5
11	1C	TDH (no pin)	Metaphysis	ATCC 12600 biofilm	10
12	1C	TDH (no pin)	Metaphysis	ATCC 25923 biofilm	10
13	2	TDH-pin	Metaphysis	Sterile saline	10
14	2	TDH-pin	Metaphysis	*S. aureus* (ATCC 12600)	10
15	2	TDH-pin	Metaphysis	*S. aureus* biofilm (ATCC 25923)	10
16	2	TDH (no pin)	Metaphysis	*S. aureus* (ATCC 12600)	10
17	2	TDH (no pin)	Metaphysis	*S. aureus* biofilm (ATCC 25923)	10
18	2	NIS-pin	Metaphysis	Sterile saline	10
19	2	NIS-pin	Metaphysis	*S. aureus* (ATCC 12600)	10
20	2	NIS (no pin)	Metaphysis	*S. aureus* biofilm (ATCC 25923)	10
Total					150

### 2.3 Study design

The animals were randomized in groups (summarized in Table 1). Three preliminary studies were carried out (1A: trialing bone defect site location; 1B: trialing alternate surgical methods; 1C: trialing a high-biofilm-forming *S. aureus* variant), and a larger study comparing surgical approaches was then undertaken (comprehensive testing of the tibial drilled hole, TDH, and the needle insertion surgery, NIS, model variants).

### 2.4 Surgery and anesthesia

Analgesia was provided by injecting buprenorphine (0.1 mg kg ${}^{-1}$ ) subcutaneously 1 h before surgery and post-surgery as required. Anesthesia was induced using ketamine (75 m kg ${}^{-1}$ ) and xylazine (10 mg kg ${}^{-1}$ ) by intraperitoneal injection, and animals were maintained on inhaled isoflurane (2 %–3 % per 1.5–2 L oxygen). The right leg of each animal was shaved and cleaned with a povidone-iodine solution prior to surgery.

For TDH surgery, a medial parapatellar approach was used to access the right proximal tibia. A hole (0.5 mm in diameter) was made at the right tibial metaphysis (below the growth plate) using a surgical drill (Stryker ^®^5100-15-250 Straight, Kalamazoo, USA), exposing the medullary canal adjacent to the drilled hole for bacterial infection. For the pin insertion, a 38mm×0.25mm stainless-steel pin (Australian Entomological Supplies Pty Ltd, South Murwillumbah, Australia) was inserted through the subchondral bone at the knee, adjacent to the hole defect. For NIS, a 25G needle was manually twisted to puncture the cortical bone of the metaphysis, and the stainless-steel pin inserted through the hole into the medullary canal (Videos 1 and 2). For local inoculation, bacterial suspension was injected directly into the defect with a Hamilton needle and syringe (Hamilton Company, Nevada, USA) immediately after pin insertion. For the hematogenous systemic infection groups, bacterial suspension was administered into the bloodstream via the lateral tail vein.

Baseline radiographs were taken at the time of surgery after the drill hole or pin insertion to confirm the drill hole and pin positions. Readjustment was carried out before closing the incision to minimize complications (e.g., pin slip and fracture). The incision was closed with 5-0 VICRYL (Ethicon LLC, Puerto Rico, USA), and no dressings were applied to the wound. Animals were allowed to recover on a heated pad after surgery and given saline subcutaneously (1 mL) to aid in rehydration, and analgesia was maintained for at least the first 72 h.

### 2.5 X-ray and postsurgical monitoring

Animals were monitored daily by experienced staff and had twice-weekly radiographs performed under anesthesia (inhaled isoflurane) using digital X-ray (Faxitron Bioptics, Tucson, AZ, USA) at 25 kV for 5 s with ×2 magnification. X-ray images were assessed by expert researchers and a facility veterinarian; all were blinded to treatment. The Mouse Grimace Scale (MGS) was also used to score the animals for monitoring pain and severity. As an ethical end point, animals showing overt physiological and/or radiological evidence of localized or systemic infection judged by declining overall health (loss of body weight, lethargy, pyrexia, poor coat condition, non-weight bearing, and inflammation of the surgical site) and/or radiological evidence of persistent infection (local osteolysis at the tibia joint) were euthanized. The remaining mice were euthanized 2 weeks postoperatively.

### 2.6 Specimen collection and bacterial assays

Blood samples were taken immediately after euthanasia via cardiac puncture, and swabs were taken from the bone (drilled hole), soft tissue adjacent to the drilled hole, and the metal pin. Pus (if present) was also collected by swab. Blood and swabs were immediately agitated in 1 mL sterile Luria–Bertani (LB) broth and cultured overnight at 37 ${}^{\circ }$ C. They were reported as either positive (turbid) or negative (clear) and then quantified using a spectrophotometer (SpectraMax^®^ iD3, Molecular Devices, San Jose, USA) at 600 nm. A positive infection was defined by a positive bacterial culture from the bone swab and pin swab with an absorbance (OD ${}_{600}$ ) >0.1 . The right tibiae were harvested for radiographic and histological studies.

### 2.7 Radiographic analysis

Tibiae were fixed in 10 % formalin for 24 h and transferred to 70 % ethanol before being scanned with a SkyScan 1272 micro-computed tomography (micro-CT) scanner (SkyScan, Kontich, Belgium). All samples were scanned in 70 % ethanol-soaked delicate task wipes (Kimwipes^®^) at 50 kV and 200 µ A using a 0.5 mm aluminum filter with 2500 ms of exposure. Images were scanned at a pixel resolution of 10 µ m, reconstructed with NRecon, straightened using DataViewer, and analyzed with CTAn software (SkyScan). A global threshold to define bone tissue was set at 0.4 g cm ${}^{-3}$ calcium hydroxyapatite, calibrated using two phantom samples of known density. Bone morphometric outcomes included bone volume (mm ${}^{3}$ ), tissue volume (mm ${}^{3}$ ), and bone tissue mineral density (g cm ${}^{-3}$ ). Three-dimensional reconstructions were generated using CTVox software (SkyScan).

### 2.8 Paraffin histology

After micro-CT scanning, the specimens were stored in 70 % ethanol until ready for decalcification. The tibiae were decalcified in 0.34M EDTA (ethylenediaminetetraacetic acid, pH 8.0) solution at 4 ${}^{\circ }$ C on a shaker for 2 weeks, with solution changes twice weekly. Following decalcification, samples were embedded in paraffin and sectioned coronally through the tibial drilled hole at a thickness of 5 µ m. Mounted sections were stained with hematoxylin and eosin (H&E), scanned digitally using the Aperio CS2 digital pathology slide scanner (Leica Biosystems, Wetzlar, Germany), and reviewed with Aperio ImageScope software.

### 2.9 Statistical analyses

Statistical power calculations and analyses were performed using GraphPad Prism (GraphPad Software, La Jolla, CA, USA), and the cutoff for significance for all tests was set to p<0.05 . In vivo studies were powered to infection rate and were compared using Fisher's exact test. The micro-CT data were analyzed with Dunn's and Kruskal–Wallis tests.

## 3 Results

### 3.1 TDH surgery led to fracture when holes were generated in the tibial midshaft

In Study 1A, it was speculated that infection risk may differ between the trabecular bone of the metaphysis and the cortical bone of the diaphysis. Following the creation of a 0.5 mm diameter drilled hole by tibial drilled hole (TDH) surgery, animals were locally inoculated with *S. aureus*. No fractures occurred within the metaphyseal defects; however, 47 % (7 of 15) midshaft (diaphyseal) defects led to premature tibial fracture within 24 h. For the metaphyseal defects, few animals showed evidence of persistent orthopedic infection (0 %–10 %), despite inoculation with *S. aureus*.

**Figure 1:**
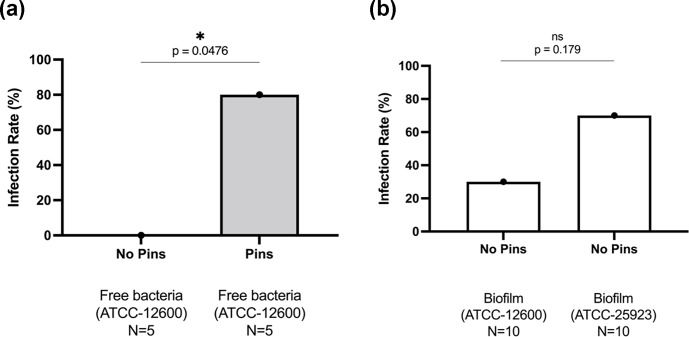
Study 1B. The bone infection rates (%) in mice that received TDH surgery and *S. aureus * inoculation: **(a)** comparison of inoculation with free ATCC 12600 bacteria with and without metal pins; **(b)** comparison of inoculation with biofilm suspensions of ATCC 12600 and ATCC 25923 without metal pins. The p values were determined by Fisher's exact test from individual experiments.

### 3.2 Metal surfaces and biofilms are essential to achieve high infection rates in the TDH model

In Study 1B, an intramedullary pin was pushed through the tibial plateau into the intramedullary space apposed to the drill hole defect. This led to frequent infection (80 %, 4 of 5) compared with mice receiving an equivalent bacterial inoculation in the absence of a pin (0 %, 0 of 5) (Fig. 1a).

In Study 1C, *S. aureus* was cultured for a week to form a biofilm, which was scraped from the surface and directly added (in suspension) to bone defects in the absence of a pin. Biofilms were grown from the original ATCC 12600 strain as well as a second ATCC 25923 *S. aureus* clinical isolate known to produce robust biofilms. In both cases, the biofilms were able to create bone defect infection (30 % vs. 70 %, respectively), even in the absence of a metal surface (Fig. 1b).

### 3.3 NIS is a less complex procedure than TDH

**Figure 2:**
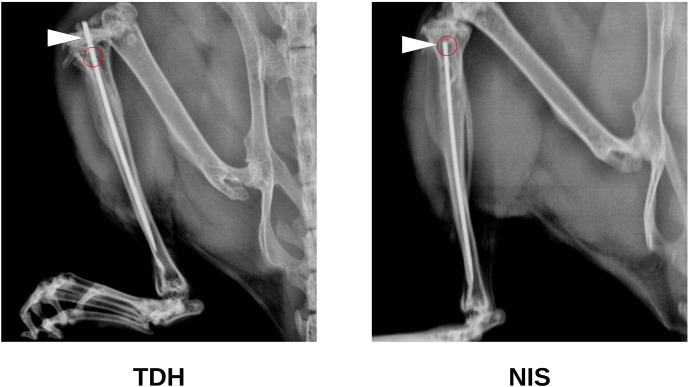
Study 1B. X-ray images illustrating the lack of disruption of the tibial condyle (white arrows) by pin insertion through the defect site (red circles) featured in the NIS model. This latter method of insertion minimized retrograde movement of the pin at the knee.

Study 1B also trialed a surgical procedure utilizing needle insertion (NIS) as an alternative to the tibial drilled hole (TDH) that did not require specialized orthopedic equipment. The NIS generated a slightly smaller hole ( <0.5 mm diameter). The metal pin was inserted via the unicortical defect rather than through the tibial condyle (Fig. 2). This contrasted with the TDH model, where insertion through the knee risked infection and/or retrograde movement of the pin (causing knee pain). Hence, the NIS procedure was gauged to be superior in these respects. Operators also reported the NIS method to be simpler and more rapid to perform. As with the TDH model, inclusion of the metal pin in the NIS model increased the infection rate compared with no pin.

### 3.4 Establishing reproducible murine models of bone defect infection

**Figure 3:**
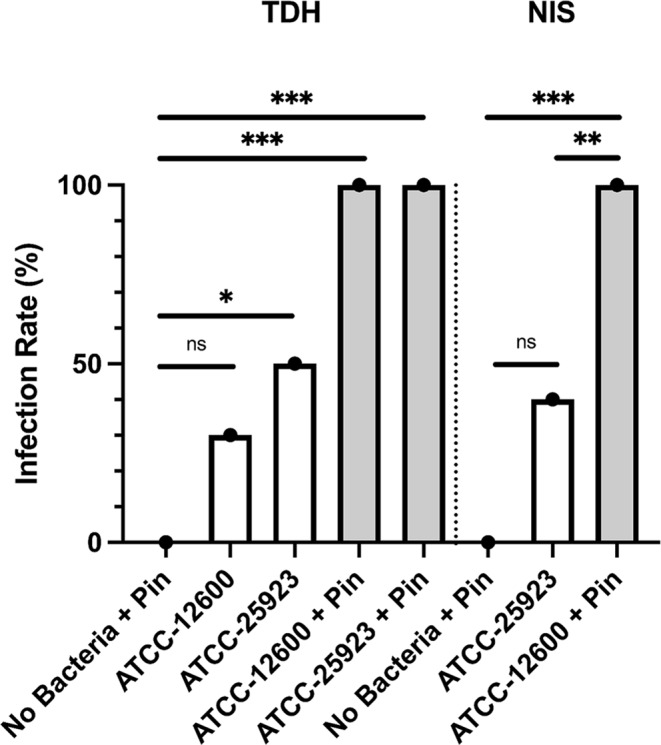
Study 2. The infection rate (%) of the TDH and NIS bone infection models inoculated with free bacteria (ATCC 12600) or biofilm (ATCC 25923) with and without metal pins. The p values are as displayed as follows: ns – no significance, ${}^{*}p\;\leq 0.05$ , ${}^{**}p\;<0.01$ , and ${}^{***}p\;\leq 0.001$ .

**Figure 4:**
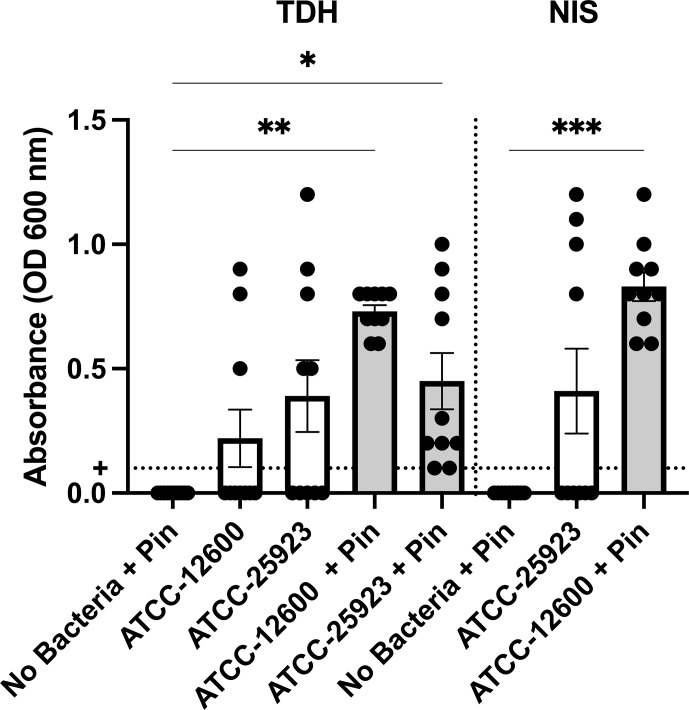
Study 2. The optical density (600 nm absorbance) readings following culture from bone swabs are shown as a surrogate measure of bacterial load. For the purposes of determining “infection” positivity or negativity, a cutoff of 0.1 was used. Significantly higher readings were seen with ATCC 12600 and a pin in the TDH and NIS models. The p values are as displayed as follows: ${}^{*}p\;\leq 0.05$ , ${}^{**}p\;<0.01$ , and ${}^{***}p\;\leq 0.001$ .

Study 2 was a large-cohort study (eight groups, N=10 per group) that addressed the impact of surgical technique (TDH and NIS models), metal implant or biofilm use, and the *S. aureus* strain (ATCC 12600 and ATCC 25923). The primary outcomes were the infection rate and bacterial load, both judged based on the optical density (600 nm) of broth cultures following a swab of the defect site. In all groups featuring inoculation and a metal pin, 100 % (10 of 10) defects were infected. Rates were lower in the absence of a pin. However, the ATCC 25923 biofilm still achieved 50 % (5 of 10) and 40 % (4 of 10) infection in the TDH and NIS models, respectively (Fig. 3). While a cutoff method was used to determine whether a specimen was infected (absorbance, ABS, 600>0.1 ), the samples remained below maximal absorbance at the time of reading; thus, the data were analyzed as a surrogate measure of bacterial load (Fig. 4). The ATCC 12600 and pin combination showed a higher load than other groups in both the TDH and NIS models. Notably, no bacteria were recovered in blood for all animals, and no evidence of infection was seen in the uninoculated negative control groups.

**Figure 5:**
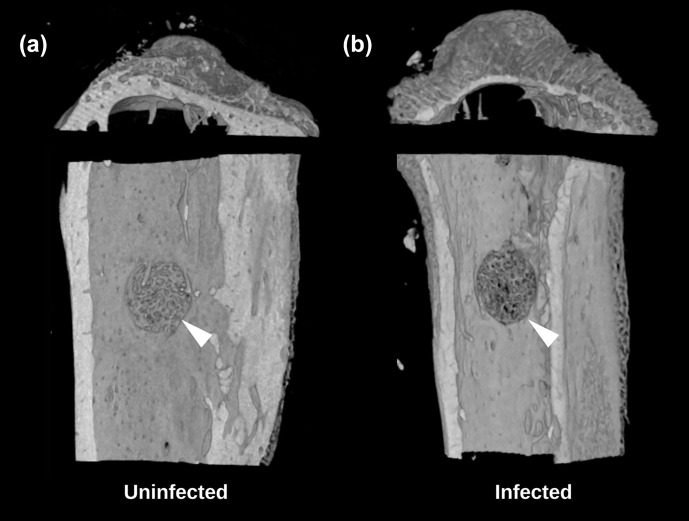
Study 2. Micro-CT reconstructions from the TDH model of the **(a)** uninfected and **(b)** infected tibia showing the reduced bone regenerate associated with infection at the defect site (white arrow).

**Figure 6:**
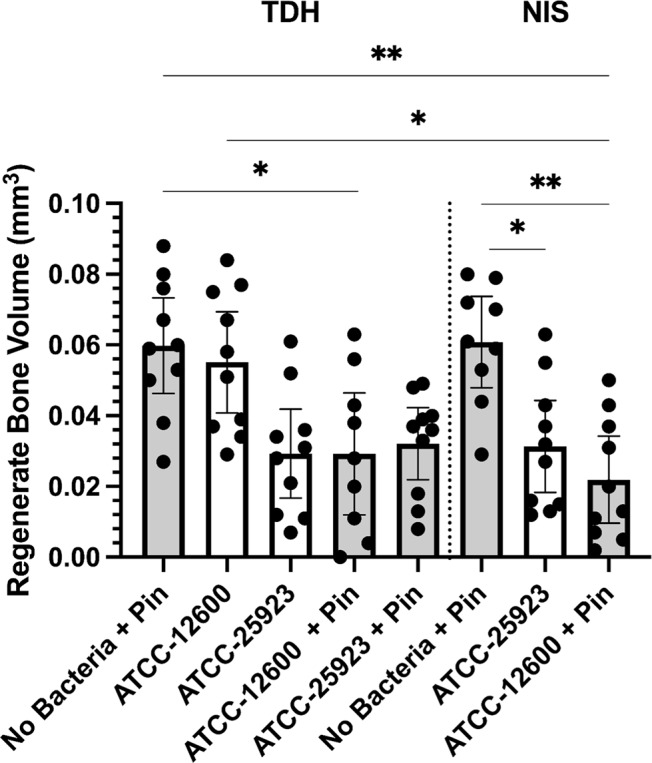
Study 2. The regenerate bone volumes (mm ${}^{3}$ ) of the tibial metaphyseal drilled hole quantified by micro-CT analysis in the TDH and NIS models. The p values are as displayed as follows: ${}^{*}p\leq 0.05$ and ${}^{**}\;p\leq 0.01$ .

A secondary outcome of the study was regenerate bone volume (BV) as quantified by micro-CT. Both the TDH and NIS models showed a reduction in regenerate bone volumes in the tibial drilled holes of the infected groups compared with the uninfected controls. The micro-CT reconstructions revealed that the infection induced a characteristic response in the bone regenerate, and there remained abundant disorganized woven bone compared with uninfected defects that had the neocortex restored (Fig. 5). The micro-CT data were quantified in terms of new regenerate in the defect (Fig. 6). ATCC 25923 biofilm infections reduced the regenerate BV by ∼50% . For injection of free ATCC 12600 bacterial infections, BV was reduced by a similar amount but only when metal pins were present.

**Figure 7:**
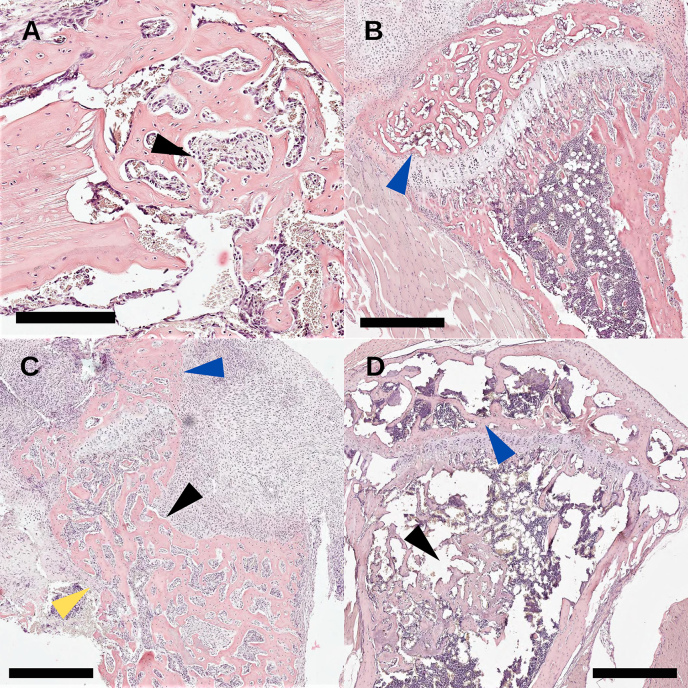
Study 2. Histology sections with H&E staining (scale bar: 500 µ m) of **(a)** an uninfected drilled hole (black arrow) with >90% healing 2 weeks post-surgery; **(b)** an uninfected tibial condyle (blue arrow) with >90 healing from pin disruption; **(c)** an infected tibia with TDH-pin insertion where severe osteolysis (back arrow) of the anterior tibia was seen, the tibial condyle was woven by reabsorption (blue arrow), and the cortical bone (yellow arrow) structure was breaking down; and **(d)** an NIS infected tibia where the tibial condyle (blue arrow) was less affected than the TDH model and the healing of the bone defect (black arrow) was incomplete.

Descriptive histology was performed on bone specimens following the TDH and NIS procedures. In the control groups that were not inoculated, the drilled hole showed abundant regeneration after 2 weeks and had no evidence of infection (Fig. 7a, black arrow). For the NIS procedure, as metal pins were inserted through the tibial plateau, the medial condyle appeared normal (Fig. 7b, blue arrow). Profound effects were seen in TDH and NIS specimens that were inoculated and went on to develop infection. Disorganized bone formation was seen concomitant with bone infection (Fig. 7c, d, black arrows), and disruption of the cortical bone often resulted in structural deformation (Fig. 7c, yellow arrow). This was the case for both the TDH and NIS models; however, the growth plate was more greatly affected by infection with the TDH model where the pin disrupted the knee (Fig. 7c vs. Fig. 7d, blue arrows).

## 4 Discussion

While many orthopedic model papers focus on the final surgical method, this report discusses the iterative approach taken to develop and refine a surgical model. It highlights some of the challenges and pitfalls associated with creating a consistent bone infection model in mice. While limited compared with rats and larger-animal models, the use of mice increases accessibility with modest housing requirements.

A notable and unexpected finding was the extremely low infection rates in the initial defect models lacking either implants or biofilm bacteria. Prior models performed by our team in rats all featured metal implants, such as intramedullary pins or titanium knee implants (Mills et al., 2018, 2020). Upon addition of a pin adjacent to the tibial defect, the high infection rates were once again achieved. This is consistent with clinical observations that implants can act as a nidus for infection. However, such implant-dependent models are restrictive for mimicking infection in the absence of an implant (e.g., Funk and Copley, 2017; Mcneil, 2020; Thakolkaran and Shetty, 2019), chronic osteomyelitis, and osteolytic lesions-associated osteomyelitis (Cha et al., 2012; Mukkamalla and Malipeddi, 2021; Gau et al., 2022). Biofilm infection emerged as an alternative method for achieving higher rates of infection in the absence of a metal pin. The use of *S. aureus* is applicable, as *S. aureus* and *S. epidermidis* are the most common bacterial pathogens associated with biofilm formation on biotic or abiotic surfaces (Schilcher and Horswill, 2020; Reffuveille et al., 2018).

We trialed two similar surgical approaches to generate a localized defect in the tibia – a drilled hole and a needle insertion defect. The TDH model uses an orthopedic drill that is comparable to those used by surgeons. However, the drill is expensive and requires specialized training to use, making it less feasible for small laboratories. In contrast, the NIS method is simpler and more cost-effective for large-scale proof-of-concepts studies. The NIS halves the surgical time by reducing the complexity of the procedure. It also makes pin repositioning easier, and it is more forgiving for minor inaccuracy. The NIS method applies less lateral force on the tibia when pushing the pin into the medullary canal, making it less likely to cause cortical bone damage and fracture. From a practical point of view, the NIS method also increases the accuracy of the hole placement by implementing the drilling manually, improving hand sensation, which is helpful for defecting a small bone. While not necessarily related to the use of a needle for defect creation, we shifted to inserting the pin through the defect hole for the NIS procedure. Disruption of the tibial condyle and the damage to the patella tendon increased the risk of complications (e.g., fracture, joint swelling, inflammation, and retrograde movement of the pin). In some cases, pin movement led to slips that had to be managed by surgical intervention or euthanasia.

This paper compares a wide range of factors speculated to affect the efficiency of the model, including bacterial pathogen, dose, surgical approach, and outcome measures. Nevertheless, the number of factors and combinations were limited based on practicality and animal numbers/ethics. To minimize the risk of sepsis, a mild static inoculation dose was used, and this never led to hematogenous infection; this is a clinical challenge in more severe cases, but one that may be ethically challenging to perform and manage in mice. Another limitation was the use of radiography and cultures as the primary methods to examine infection; future studies could employ measures such as neutrophil count, erythrocyte sedimentation rate (ESR), and C-reactive protein (CRP). The studies also were limited to *S. aureus* as a pathogen, although two different strains were tested. The model could be readily adapted for other strains including methicillin-resistant *S. aureus*, *Streptococcus* spp., *Pseudomonas aeruginosa*, and *Escherichia coli* (Lienard et al., 2021; Cassano et al., 2020; Foong et al., 2021; Pliska, 2020; Gornitzky et al., 2020). Other expanded variations that could be tested include the effects of mouse age, systemic inoculation reminiscent of a hematogenous infection, and the ability to model chronic osteomyelitis (Alstrup et al., 2021; Billings and Anderson, 2022; Jensen et al., 2017; Joyce et al., 2021; Lüthje et al., 2020; Roux et al., 2021).

## 5 Conclusions

This report describes the iterative development of bone defect infection models. Detailed methods (Protocols in the Supplement) are provided for the NIS-pin and NIS-biofilm orthopedic murine models based on the outcomes of Study 2. Both models are considered to have different utilities – for modeling implant- and non-implant-associated infection, respectively. These simplified and cost-effective methods are suitable for conducting preclinical trials and proof-of-concept studies for interventions to prevent and treat osteomyelitis.

## Supplement

10.5194/jbji-8-81-2023-supplement The supplement related to this article is available online at: https://doi.org/10.5194/jbji-8-81-2023-supplement.

## Data Availability

Data are available from the corresponding author upon request.

## Video supplement

Video 1: demonstration of the needle insertion surgical technique (, Dao, 2023a); Video 2: demonstration of the drilled hole surgical technique (, Dao, 2023b). Active links to the videos are also available from the corresponding author upon request.
