# Supplementary material for: Murine models of orthopedic infection featuring Staphylococcus aureus biofilm
Source: J Bone Jt Infect. 2023 Mar 7;8(2):81–9. doi: 10.5194/jbji-8-81-2023 (PMC10134754; doi:10.5194/jbji-8-81-2023)
Supplement: The supplement related to this article is available online at: https://doi.org/10.5194/jbji-8-81-2023-supplement. [file jbji-8-81-supplement.pdf]

Supplement of J. Bone Joint Infect., 8, 81–89, 2023
https://doi.org/10.5194/jbji-8-81-2023-supplement
© Author(s) 2023. CC BY 4.0 License.

Supplement of

Murine models of orthopedic infection featuring *Staphylococcus aureus* biofilm

Aiken Dao et al.

Correspondence to: Aaron Schindeler (aaron.schindeler@sydney.edu.au)

The copyright of individual parts of the supplement might differ from the article licence.

Supplementary protocol

Based on our research findings, we proposed the supplementary methods of the needle-insertion surgery (NIS) to induce osteomyelitis in murine. These protocols should increase the reliability and reproducibility of the model for preclinical trials.

1. Materials

1.1. Animals

For the NIS-biofilm and NIS-pin bone infection models, we recommend using female C57BL/6 mice at the age of 8-12 weeks. However, other mouse strains, age and gender can be amended for the need of the experiment. Genetically modified (GM) mice are also suitable for this surgery, however, there may be additional risk of fracture and other complication if the phenotype reduces the bone mineral density or mechanical feature of the animal bone.

1.2. Bacterial strains

For general studies, we strongly recommend using a high biofilm forming *S. aureus* strains (e.g., ATCC-25923) for the NIS models. If other pathogens are being tested, a biofilm-forming strain of that pathogen should be employed.

1.3. Lysogeny broth (LB)

1. Mix tryptone powder (5 g), yeast extract (2.5 g), sodium chloride (5 g) and dH2O water (500 mL) in a reagent media bottle.
2. Autoclave the dissolved medium at 15 lbs pressure (121°C) for 15-30 minutes.

1.4. Lysogeny broth agar (LB agar)

1. Mix tryptone powder (5 g), yeast extract (2.5 g), sodium chloride (5 g), agar A (7.5 g) and MilliQ water (500 mL) in a reagent media bottle.
2. Autoclave the dissolved medium at 15 lbs pressure (121°C) for 15-30 minutes.
3. Pour the agar (10 mL) to a Petri dish and let it cool down at room temperature using antiseptic technique.

1.5. Tryptic Soy Broth (TSB) + 10% glucose (for NIS-biofilm model)

1. Mix 15 g of tryptic soy broth power (Mediatech Inc, Manassas, VA, Germany) with 1.5 g of glucose and 500 mL of MiliQ water.
2. Autoclave the dissolved medium at 15 lbs pressure (121°C) for 15-30 minutes.

1.6. Drugs

- Ketamine (75 mg/kg)
- Xylazine (10 mg/kg)
- Buprenorphine (0.1 mg/kg)
- Isoflurane (2-3% per 1.5-2L oxygen)

1.7. Surgical equipment

1.7.1. NIS-biofilm model

- Scalpel handle #3
- Surgical blade #15
- Needle holder
- Hamilton syringe and needle
- 25G needle
- 5-0 Vicryl Rapide ® coated sutures
- Isoflurane Vaporizer with a nose cone

1.7.2. NIS-pin model

- Scalpel handle #3
- Surgical blade #15
- Needle holder
- Hamilton syringe and needle
- 25G needle
- Stainless-steel pin (0.5 mm diameter/size 000)
- Wire pliers
- 5-0 Vicryl Rapide ® coated sutures
- Isoflurane Vaporizer with a nose cone

2. Methods

2.1. Free bacterial culture (for NIS-pin model)

1. Streak the bacterial culture (e.g., ATCC-25923 *S. aureus*) from glycerol stock –80°C) onto a lysogeny broth (LB) agar plate.
2. Incubate the plate at 37°C overnight.
3. From the streaked plate, pick a single colony and resuspend it in 1 mL of LB broth in a 10 mL falcon tube.
4. Incubate at 37°C overnight on a shaker.
5. Quantify the bacteria with a spectrophotometer at 600 nm (absorbance of 0.3 should be equivalent to 3×10^8 CFU/mL).
6. Perform a serial 10-fold dilution in injectable saline (0.9% sodium chloride) to reach a bacterial concentration of 10^6 to 10^7 colony forming unit (CFU) for inoculation.

Also see Figure S1.

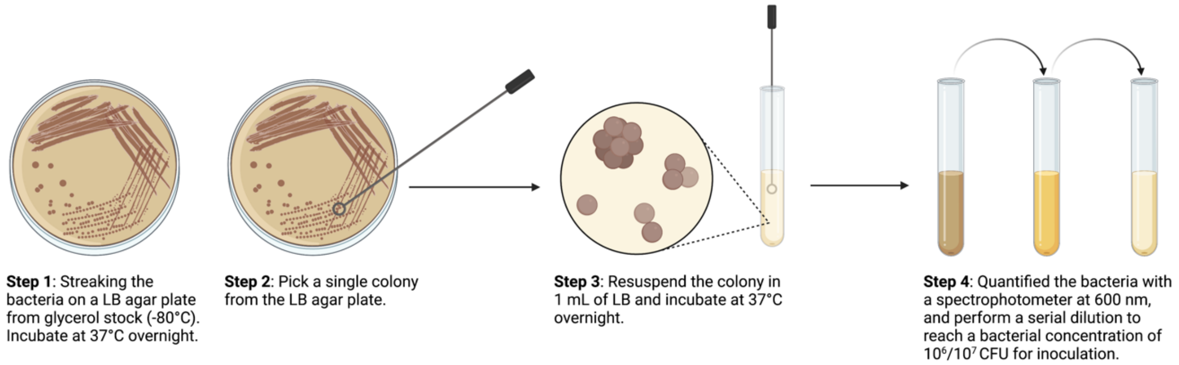

Figure S1: The protocol for free bacterial culture for local inoculation.

Note: the bacterial concentration is adjustable with serial dilution. For NIS-Pin model, the bacterial concentration can be as low as 10^4 to 10^5 CFU for inoculation.

2.2. Bacterial biofilm culture (for NIS-biofilm model)

1. Streak the bacterial culture (e.g., ATCC-25923 *S. aureus*) from glycerol stock (–80°C) onto a lysogeny broth (LB) agar plate.
2. Incubate the plate at 37°C overnight.
3. From the streaked plate, pick a single colony from the streak plate and resuspend it in 1 mL of LB broth.
4. Incubate at 37°C overnight on a shaker.
5. Place a piece of sterile stainless-steel/titanium foil (1 cm × 1 cm) in a well of a 24-well plate.
6. Pipette 100 μL bacteria into the well.
7. Add 900 μL of tryptic soy broth (TSB) with 10% glucose to the well.
8. Incubate at 37°C for 5-7 days (change the media daily).
9. To change the media, pipette out all the media without disturbing the foil, and add fresh 1 mL of TSB with 10% glucose to the well.
10. Scrape the biofilm off the foil with a sterile swab
11. Resuspend the biofilm in 1 mL of normal saline.
12. Quantify the biofilm with a spectrophotometer at 600 nm (absorbance of 0.3 should be equivalent to 3 × 10^8 CFU/mL).
13. Perform a serial 10-fold dilution to reach a biofilm concentration of 10^6 to 10^7 colony forming unit (CFU) for inoculation.

Also see Figure S2.

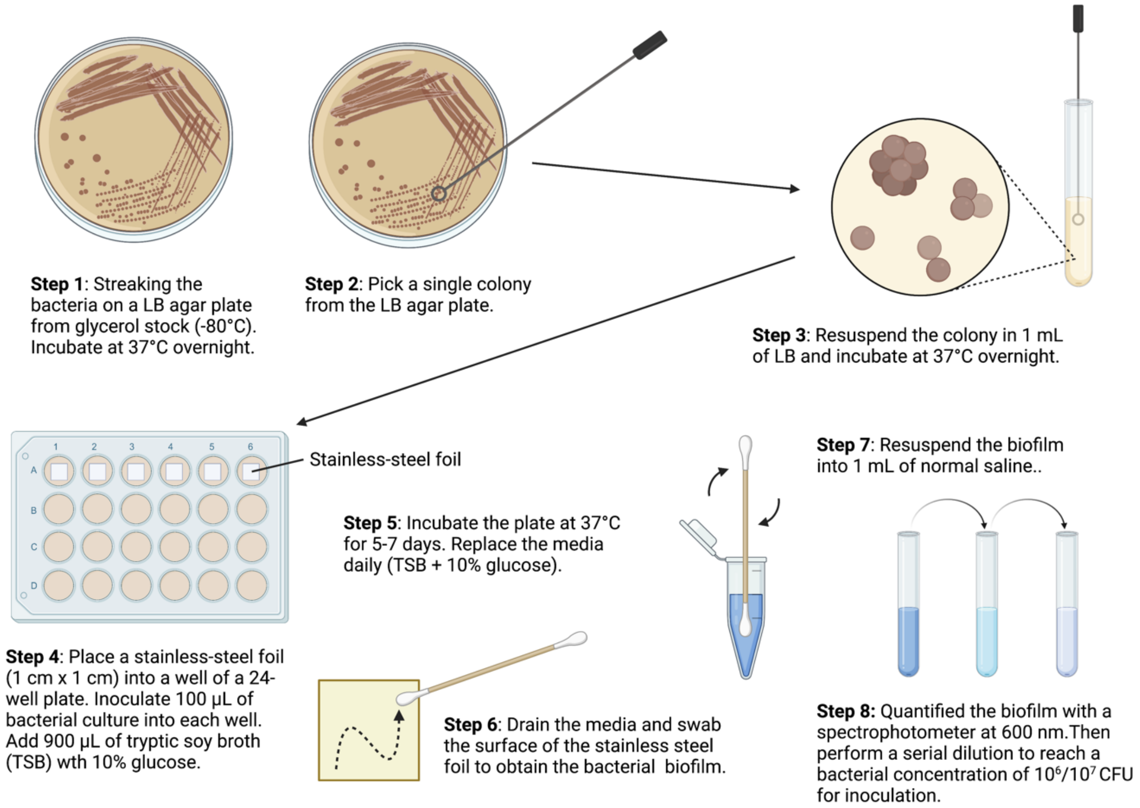

Figure S2: The protocol for free bacterial culture for local inoculation.

Note: the bacterial concentration is adjustable with serial dilution. For NIS-Pin model, the bacterial concentration can be as low as 10^4 to 10^5 CFU for inoculation.

2.3. Pain relief, anesthesia and preparation

1. Give 0.1 mg/kg buprenorphine or pain relief subcutaneously (SC) at least one hour before surgery.
2. Before surgery, inject ketamine (75m/kg) and xylazine (10mg/kg) by intraperitoneal (IP) injection for anesthesia.
3. Shave the animal's right leg.
4. Sterilize the surgical area with a povidone-iodine solution.
5. If required, give isoflurane (2-3% per 1.5-2 L oxygen) through a nose cone during the procedure to maintain anesthesia.
6. Check the paw pinch reflex before incision.

2.4. NIS-biofilm model

1. Make a medial parapatellar incision to access the right proximal tibia.
2. Use a 25G needle to drill a hole on the tibial metaphysis (below the growth plate), exposing the medullary canal.
3. Inject 5 μL of 10^{6-7} CFU of biofilm suspension with a Hamilton syringe into the drill hole.
4. Perform a full/lower-body XR (25 kV for 15 seconds) scan to examine the drilled hole.
5. Close the incision with 5-0 Vicryl Rapide ® coated sutures.
6. Apply no dressings to the wound.

Also see Figure S3 and Video 1.

Note: since biofilm alone does not induce osteomyelitis very efficiently without a metal surface, a higher dose (>10^7 CFU) can be used to increase the infection rate if 100% infection rate is not achieved.

2.5. NIS-pin model

1. Make a medial parapatellar incision to access the right proximal tibia.
2. Use a 25G needle to drill a hole on the tibial metaphysis (below the growth plate), exposing the medullary canal.
3. Inject 5 μL of 10^5 CFU of biofilm suspension with a Hamilton syringe into the drill hole.
4. Insert a stainless-steel pin through the drilled-hole defect into the medullary canal of the tibia until it reaches the end of the canal.
5. Band the pin at 90° to prevent the pin from slipping out.
6. Cut the pin off with the wire pliers
7. Perform a full/lower-body XR (25 kV for 15 seconds) scan to monitor the drilled hole.
8. Close the incision with 5-0 Vicryl Rapide ® coated sutures.
9. Apply no dressings to the wound.

Also see Figure S4 and Video 2.

Note: since free bacteria induce osteomyelitis efficiently when a metal surface is present, a lower dose ($<10^5$ CFU) can be used to reduce the severity of the bone infection.

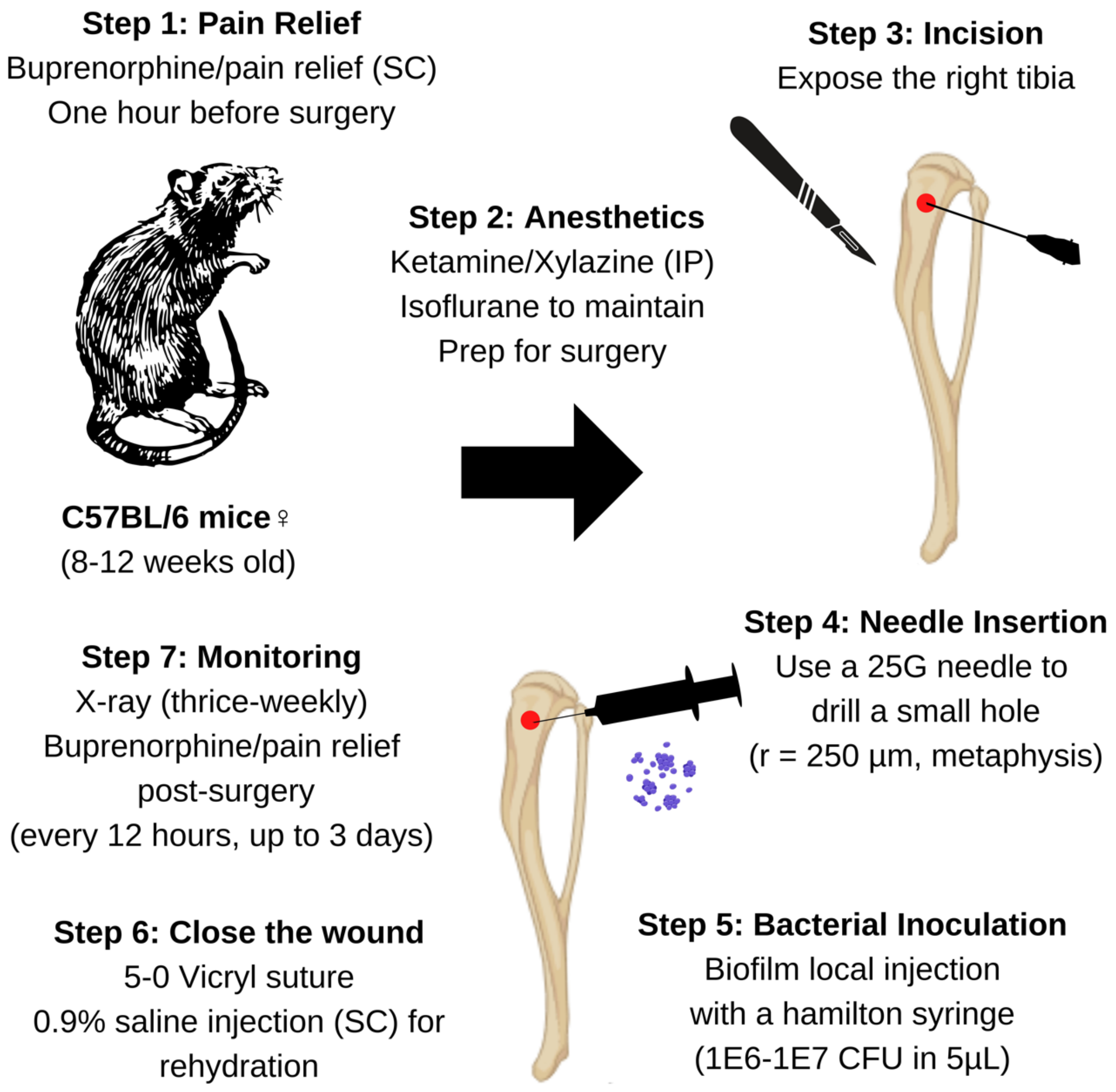

Figure S3: The protocol for the NIS biofilm bone infection model.

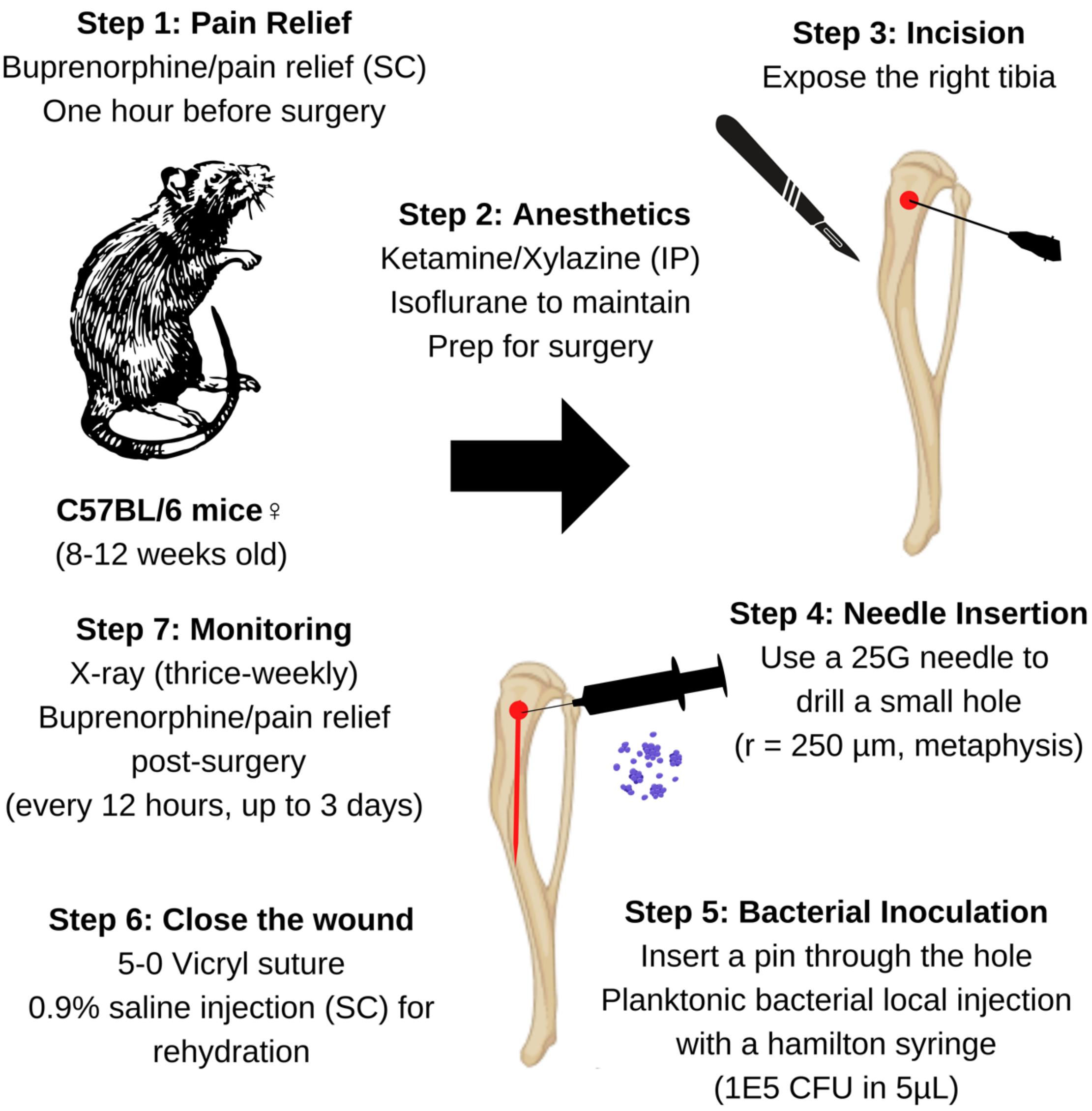

Figure S4: The protocol for the NIS-pin bone infection model.

2.6. Post-Surgical Recovery

1. Inject 1 mL of saline (0.9% sodium chloride) subcutaneously into the mouse for rehydration.
2. Let the animal recover within a box/cage on a heating pad with soft bedding for 30 mins or until it recovers from anesthesia.
3. Monitor breathing and heart rate every 3-5 minutes.
4. If the breathing is too shallow and slow or the heart rate is too rapid, provide 100% oxygen. (Normal HR: 310-840 beats/min and RR: 80-230 breaths/min for mice)
5. Check for righting reflex and motor recovery.

2.7. Post-Surgical Monitoring and Pain Relief

1. Give buprenorphine subcutaneously every 12 hours (for at least three days). (Note: pain relief should also be given when the animal is in pain, use Mouse Grimace scales to evaluate animals for pain).
2. Perform a lower-half body XR scan (25 kV for 15 seconds) thrice weekly to monitor the infection progression and complication (e.g., osteolysis, fracture).
3. Other forms of pain relief (approved by ethics) can substitute buprenorphine to minimize its side effects.
